# Predictive factors for non-small-volume central lymph node metastases (more than 5 or ≥ 2 mm) in clinically node-negative papillary thyroid carcinoma

**DOI:** 10.1097/MD.0000000000014028

**Published:** 2019-01-04

**Authors:** Jian-Biao Wang, Ya-Yu Sun, Liu-Hong Shi, Lei Xie

**Affiliations:** a Departments of Head and Neck Surgery; b Diagnostic Ultrasound and Echocardiography, Institute of Micro-Invasive Surgery of Zhejiang University, Sir Run Run Shaw Hospital, School of Medicine, Zhejiang University, Hangzhou, Zhejiang, P. R. China.

**Keywords:** central lymph node metastasis, non-small-volume central lymph node metastases, papillary thyroid carcinoma, prophylactic central neck dissection, risk factors

## Abstract

The benefits of prophylactic central neck dissection (pCND) for treating patients with clinical node-negative (cN0) papillary thyroid carcinoma (PTC) remain controversial. Lymph node metastases have been strongly associated with local recurrence and low survival, especially in PTC patients with 5 or more or ≥2 mm metastatic lymph nodes. The following study investigates the incidence and risk factors of more than 5 or ≥2 mm metastatic lymph nodes in the central compartment.

A total of 611 patients with cN0 PTC were retrospectively analyzed. Cervical lymph nodes were harvested, and the size of metastatic lymph nodes was consequently analyzed.

Non-small-volume central lymph node metastases (NSVCLNM), defined as more than 5 or ≥2 mm metastatic lymph nodes) were detected in 67 (11.0%) patients. Male gender, age ≤36 years, multifocal lesions, extrathyroidal extension, and tumor size > 0.85 cm were independent risk factors for NSVCLNM in cN0 PTC. The sensitivity and specificity of having ≥3 risk factors for predicting NSVCLNM was 46.3% and 86.8%, respectively.

These findings suggest that pCND is a suitable treatment strategy for cN0 PTC patients with 3 or more risk factors for NSVCLNM.

## 1 Introduction

Cervical lymph node metastasis (LNM) is relatively common in patients with papillary thyroid carcinoma (PTC).^[1,2]^ Even in patients with clinically node-negative (cN0) PTC, LNM is found in 60.9% of the central lymph nodes.^[3]^ LNM has been associated with local recurrence and low survival,^[4–6]^ especially in PTC patients with 5 or more^[7]^ or ≥2 mm metastatic lymph nodes.^[8]^ Cranshaw et al have demonstrated that the risk of lymph node recurrence in patients with metastatic lymph node ≥2 mm is significantly higher compared to patients with histologically proved micrometastases.^[8]^ In addition, Sugitani and colleagues have reported that the risk of recurrence is significantly higher in patients with 5 or more LNM (19%) compared to those having <5 metastases (8%).^[7]^

The application of prophylactic central neck dissection (pCND) during primary surgery for cN0 PTC remains controversial. The possible treatment benefits of pCND should be weighed against the potential procedure related risks.^[9]^ Indeed, several studies have documented greater morbidity rates, such as transient hypoparathyroidism, for patients undergoing central neck dissection (CND) with total thyroidectomy (TT) compared to those who received TT alone.^[10,11]^ Therefore, there is an urgent need to investigate an optimal treatment protocol for pCND in order to balance the benefits and risks of the surgery.

According to the American Thyroid Association (ATA) modified 2009 risk stratification system, low-risk PTC patients (<5% risk of recurrence) are those with small-volume LNM (clinical N0 or ≤5 pathologic N1 micrometastases, <2 mm in largest dimension),^[12]^ while those with non-small-volume central lymph node metastases (NSVCLNM; defined as more than 5 or ≥ 2 mm metastatic lymph nodes) are considered as high-risk group.^[12]^ Patients with high-risk for recurrence are considered as good candidates for pCND due to the higher benefit of this therapy (higher risk and higher benefit from the intervention). Ensuring the predictive factor of NSVCLNM in cN0 PTC patients could suggest a more selective approach to identify patients with the necessity of pCND.

In this study, we investigated the frequency and predictive factors of NSVCLNM in cN0 PTC patients, using a large group of Chinese patients.

## 2 Patients and methods

### 2.1 Patients

A retrospective analysis was performed based on a prospectively collected database collected from the Head and Neck Surgery Department in Sir Run Run Shaw Hospital, Medical School of Zhejiang University. The study cohort included 611 consecutive cN0 patients who were scheduled to undergo standard open transcervical thyroidectomy for primary PTC between January 2013 and December 2015. The inclusion criteria were the absence of previous thyroid surgery, cN0 neck, and availability of medical history information. Patients with incidental PTC during thyroidectomy for benign conditions (without neck dissection) were excluded from the study. A cN0 neck was defined as neck with no palpable cervical lymph nodes and no suspicious metastatic nodes diagnosed using ultrasonography and computed tomography (CT).

Preoperative neck ultrasound was used to evaluate thyroid, as well as central and lateral neck lymph nodes in each patient. Ultrasound-guided fine needle aspiration (FNA) was used to confirm malignancy or metastasis of suspicious primary lesions in the lateral cervical lymph nodes. CT was conducted from the mastoid of the temporal bone to the aortic arch in patients with lateral neck LNM. Vocal cord function was assessed by direct or indirect laryngoscopy. In addition, the thyroid hormones, parathyroid hormone, calcitonin, and serum calcium was also measured in each patient.

In accordance with the guidelines of ATA (2009),^[13]^ TT was performed if the patient met one of the following criteria: bilateral nodularity, extrathyroidal extension, tumor diameter > 1.0 cm, multifocal lesions in the affected lobe, and regional or distant metastases. Thyroid lobectomy was performed for small (<1 cm), low-risk, unifocal, and intrathyroidal papillary carcinomas in the absence of prior head and neck irradiation, or radiologically or clinically involved cervical node metastases. Ipsilateral CND was performed routinely, whereas bilateral CND was applied if the lesions, which occurred in the isthmus or the malignant nodules were located in both lobes. The modified lateral neck dissection, including levels II–IV, was performed only in patients with clinically evident nodal disease on preoperative US or when the FNA of a lateral node was positive.

The Ethical Committee of Sir Run Run Shaw Hospital, Medical School of Zhejiang University, approved this study.

### 2.2 CND surgical procedure

All the procedures included conventional open surgeries and were performed by the same panel of senior surgeons. Thyroidectomy was performed using the technique of capsular dissection suggested by Thompson et al.^[14]^ Recurrent laryngeal nerves and all the parathyroid glands were routinely identified and were preserved under direct vision. The vascular supply of the parathyroid glands was confirmed by the fine needle pricking test. Devascularized parathyroid gland was excised into tiny fragments and was autotransplanted into the contralateral sternocleidomastoid muscle.

Based on ATA guidelines, bilateral CND entails the removal of the prelaryngeal, pretracheal, and both the right and left paratracheal nodal basins; while, unilateral CND involves the removal of the prelaryngeal, pretracheal, and the single paratracheal nodal basin.^[15]^

In this study, the central compartment lymph nodes were collected and analyzed. The number of metastatic lymph nodes was based retrospectively on the initial pathological reports. Specifically, all metastatic lymph nodes foci were microscopically analyzed by 1 pathologist who prospectively reviewed the metastatic lymph nodes. In cases where multiple metastatic foci were observed, the largest one was recorded as the size of metastatic lymph node. The NSVCLNM was defined as metastatic lymph node more than 5 or ≥2 mm in size, located in the central neck compartment.

### 2.3 Clinicopathological factors

The following clinicopathological factors associated with NSVCLNM were documented: sex, age, coexistent thyroid disease, maximal tumor size, extrathyroidal extension, and multifocal/solitary lesions. Extrathyroidal extension was evaluated based on intraoperative findings and frozen section analysis. Maximal tumor size and multifocal/solitary lesions were both determined through paraffin section pathological examination.

### 2.4 Statistical analysis

Statistical analyses were performed using SPSS version 16.0 (IBM, Armonk, New York). Continuous data are present as mean (s.d.) or median (range). Baseline patient characteristics were compared between the groups using Student *t* test or Mann–Whitney *U* test for continuous variables, and Pearson χ^2^ test for categorical variables. A receiver-operating characteristic (ROC) analysis was used to identify the cut-off point of the primary tumor size and age for defining the risk of NSVCLNM. The odds ratio (OR) and 95% confidence interval (CI) for relationships between each variable and NSVCLNM (yes/no) were calculated using binary logistic regression. *P* <.05 was considered statistically significant.

## 3 Results

### 3.1 Patient characteristics

Among 611 patients who underwent initial thyroidectomy for PTC, there were 144 men and 467 women with a mean age of 43.7 years (range, 13–78 years). All patients were diagnosed with primary PTC by frozen section examination intraoperatively. In addition, 156 patients had Hashimoto's thyroiditis. Overall, three different surgical procedures were performed:

(1) lobectomy (including isthmectomy and pyramidal lobectomy) with ipsilateral CND (right 114 patients and left 100 patients);
(2) TT with ipsilateral CND (right 120 patients and left 119 patients); and
(3) TT with bilateral CND (158 patients).

The median number of harvested central lymph nodes was 11.0 (range 1–66), and the median number of metastatic central lymph nodes was 0.0 (range 0–17). Central lymph node metastases (CLNM) were detected in 229 (37.5%) patients, while NSVCLNM were found in 67 (11.0%) patients.

None of the patients had the history of head and neck radiation before surgery, and none of the patients had distant metastasis. The characteristics of these patients are listed in Table 1.

**Table 1:**
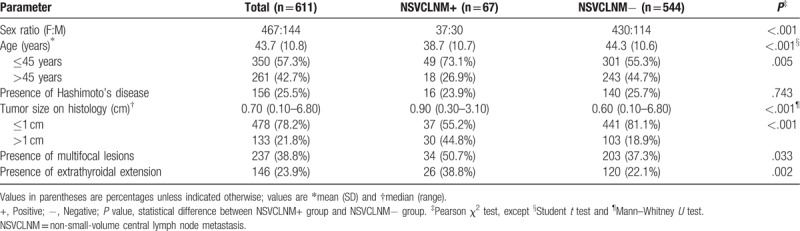
Patient demographics.

### 3.2 Risk factors for NSVCLNM in cN0 PTC

**Figure 1:**
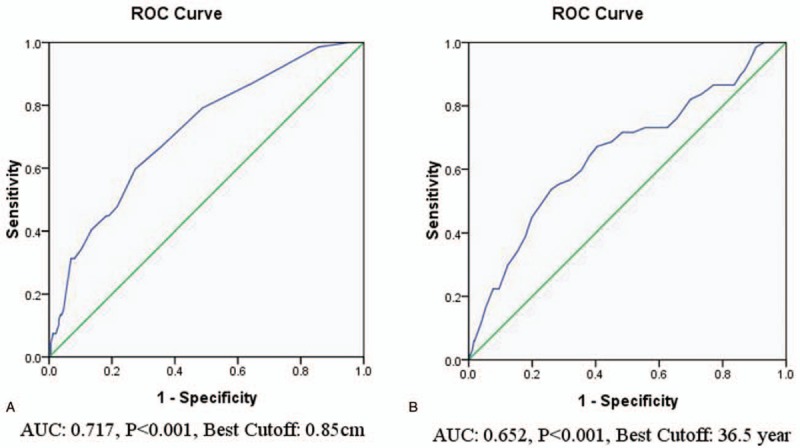
ROC curve analyses of the cutoff point for size of the primary tumor (A) and age (B) in predicting NSVCLNM in cN0 PTC. NSVCLNM = non-small-volume central lymph node metastasis, PTC = papillary thyroid carcinoma, ROC = receiver-operating characteristic.

ROC curve analysis was used to determine the cut-off point for size of the primary tumor (0.1–6.8 cm) for predicting the risk factors of NSVCLNM in patients with cN0 PTC. A tumor size of 0.85 cm was found to be the cut-off point for NSVCLNM (Fig. 1A). Tumor size greater than 0.85 cm was significantly associated with NSVCLNM according to univariate and multivariate logistic regression analyses (Table 2).

**Table 2:**
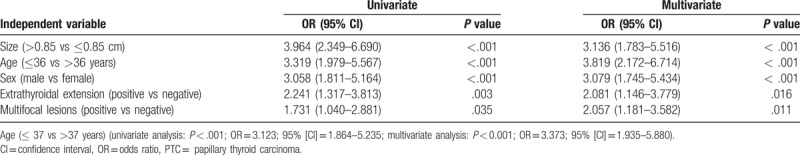
Univariate and multivariate logistic regression analyses of NSVCLNM in cN0 PTC patients.

Additionally, NSVCLNM was mainly observed in younger patients (Table 1). Consequently, ROC curve analysis was used to determine the optimal age cut-off point (range, 13–78 years) for predicting NSVCLNM in patients with cN0 PTC (Fig. 1B). Age's ≤36 years and ≤37 years were both significantly associated with NSVCLNM according to the univariate logistic regression, but the former had a higher OR and 95% CI (Table 2).

Other variables, such as male gender, extrathyroidal extension, and multifocal lesions were also significantly associated with NSVCLNM based on univariate logistic regression. Conversely, concomitant Hashimoto's thyroiditis had no significant influence on NSVCLNM (*P* = .743). Multivariate logistic regression analysis confirmed that male gender, age ≤36 years, multifocal lesions, extrathyroidal extension, and primary tumor size >0.85 cm were independent risk factors for NSVCLNM in patients with cN0 PTC (Table 2).

### 3.3 Scoring of risk factors for NSVCLNM in cN0 PTC

Results of multivariate analysis (Table 2) demonstrated that 5 parameters, such as male gender, age ≤36 years, multifocal lesions, extrathyroidal extension, and tumor size >0.85 cm were all independent risk factors for NSVCLNM in cN0 PTC patients with a similar level of contribution. We also investigated whether the incidence of NSVCLNM in patients with none, 1 or 2 and more of these parameters differed from one another. The patients were given a score of 0 to 5 according to the number of parameters they had. A total of 125 patients scored 0, 205 patients had score 1, 178 patients had score 2, 82 patients had score 3, 19 patients had score 4, and 2 patients had a score 5. The incidence of NSVCLNM in patients who scored 3 or more was significantly higher than in patients who scored 2 (*P* = .003); while it was also higher in patients who scored 2 compared to patients with score 1 (*P* < .001). The incidence of patients who scored 1 was similar with patients with score 0 (*P* = .776) (Table 3). The sensitivity and specificity of ≥ 3 of the above 5 variances for predicting NSVCLNM in cN0 PTC were 46.3% and 86.8%, respectively, while the positive predictive value and negative predictive value were 30.1% and 92.9%, respectively.

**Table 3:**
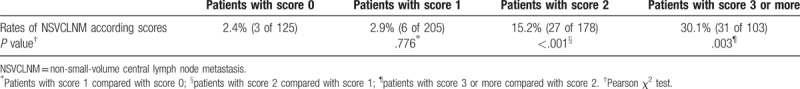
Rates of NSVCLNM according to scores.

## 4 Discussion

The benefits of pCND for treating patients with clinical node-negative PTC remain controversial. The generally indolent nature of PTC, its excellent prognosis, the operative risks of hypoparathyroidism and recurrent laryngeal nerve injury coupled with a paucity of scientific support for improved survival with pCND have led some surgeons to reserve this procedure for patients with clinically evident central compartment nodal disease. Nonetheless, several studies have suggested that the presence of cervical LNM is associated with increased nodal recurrence.^[16–18]^ In addition, pCND has shown to increase the disease-specific survival rates and decrease the rate of locoregional recurrence.^[19,20]^ Therefore, it is of great importance to identify the subsets of PTC patients who may benefit from pCND.

Recently, Randolph et al have reported that small-volume microscopic LNM in PTC are often associated with little clinical significance.^[21]^ Even though small-volume microscopic LNM were present in up to 80% of patients diagnosed with papillary thyroid microcarcinoma (PTMC), locoregional recurrence rates in treated patients ranged from 2% to 6% regardless of the extent of lymph node dissection and whether or not radioactive iodine was given as adjuvant therapy after surgical resection.^[3,22–32]^ Additionally, another recent study has indicated that patients with macroscopic PTC (primary tumor > 1 cm) have microscopic nodal disease rates of up to 62% in cN0 central neck compartment even though the recurrence rates range only from 1% to 6% if CND is not performed.^[21,33]^

In this study, we systematically analyzed the frequency and risk factors of NSVCLNM in 611 patients with cN0 PTC. To our knowledge, this is the first study concerning NSVCLNM in PTC. The prevalence of CLNM and NSVCLNM in cN0 PTC patients was 37.5% (229 of 611) and 11.0% (67 of 611), respectively.

In our study, univariate and multivariate analyses were used to evaluate the risk factors of NSVCLNM. We found that male gender, age ≤ 36 years, multifocal lesions, extrathyroidal extension, and primary tumor >0.85 cm were independent risk factors for NSVCLNM in cN0 PTC patients.

Age was an important prognostic factor for patients with PTC. Previous studies have reported that age <45 is a significant risk factor for CLNM in cN0 PTC patients.^[34,35]^ In addition, Miyauchi and his team have reported that <40 years of age is the only significant risk factor for tumor size enlargement and novel appearance of LNM of low-risk PTMC during active surveillance.^[36]^ Miyauchi's team has also found that the proportion of appearance of novel LNM in PTMC patients <40 years of age is highest (16.1%) after 10 years of observation.^[36]^ In our study, patients with NSVCLNM were significantly younger than those without NSVCLNM (38.7 [10.7] vs 44.3 [10.6] years, *P* <.001). We used ROC curve analysis to determine the cut-off age for the prediction of NSVCLNM which showed that age ≤36 years was significantly associated with an increased risk of NSVCLNM in cN0 PTC patients.

Although the incidence of thyroid cancer is higher in women compared to men, the rates of thyroid cancer-related malignancy and mortality are higher in men.^[37,38]^ What's more, male gender has been identified as a risk factor which may be suggestive of thyroid carcinoma.^[39]^ Several studies have also reported that among PTC patients, men exhibit poorer prognosis rates than women.^[40,41]^ Previous studies have demonstrated that male gender is a significant risk factor for CLNM in cN0 PTC patients.^[26,34,35,42]^ Furthermore, Nixon et al have reported that male gender is the risk factor associated with central neck node recurrence in PTC patients without pCND.^[43]^ In this study, we also found that male gender was a risk factor for NSVCLNM in cN0 PTC.

Tumor size is another important factor in TNM staging for PTC, and large tumors tend to be more aggressive.^[44]^ Several studies have shown that tumor size is significantly associated with CLNM, but the cut-off points were different. Ito^[45]^ and Sun^[34]^ have reported that tumor size >2 cm is the strongest predictor of CLNM in PTC. Bozec,^[46]^ Choi,^[47]^ and Koo^[48]^ have reported that tumor size >1 cm is associated with CLNM in PTC, while Zhang^[35]^ and Kim^[49]^ have reported that tumor sizes >6 mm and >5 mm are associated with CLNM in PTMC, respectively. In our study, tumor size in NSVCLNM patients was significantly greater than in those without NSVCLNM (0.90 [0.30–3.10] vs 0.60 [0.10–6.80] cm, *P* <.001). ROC curve analysis was used to determine the cutoff point of tumor size for predicting NSVCLNM and found that tumor size >0.85 cm was the strongest predictor of NSVCLNM in cN0 PTC.

The existence of multifocal lesions was recognized as a risk factor for CLNM in PTC^[34]^ and PMTC.^[35]^ Likewise, in the present study, multifocal lesions consistently had an effect on the risk of NSVCLNM for cN0 PTC. The incidence of extrathyroidal extension in well-differentiated thyroid cancer varies from 5% to 34%.^[50]^ Extrathyroidal extension has always been considered as an indicator of tumor aggressiveness, and a significant risk factor for LNM in PTC.^[34,35,51,52]^ In our cohort, 23.9% of the patients (146 of 611) manifested extracapsular gross and/or microscopic invasion of the primary tumor. Multivariate logistic regression analysis revealed that extrathyroidal extension was also associated with NSVCLNM in cN0 PTC.

In this study, we found that the incidence of NSVCLNM in cN0 PTC was low and was significantly lower compared to the prevalence of CLNM (11.0% vs 37.5%, *P* <.001). Small-volume microscopic LNM in PTC has often had little clinical significance.^[21]^ Cranshaw et al have demonstrated that the risk of lymph node recurrence in well-differentiated thyroid cancer patients with histologically proven micrometastases is broadly equivalent to those without LNM (5% vs 6%).^[8]^ Miyauchi has reported that after identification of biopsy-proven low-risk PTMC, the risk of developing clinically apparent LNM over a 10 years period is only 3.8%.^[36]^ Furthermore, Nixon and his team have reported that the rate of central node recurrence in PTC patients without pCND after 5 years follow-up is only 0.7%.^[43]^ Accordingly, we think that the routine performance of pCND for all cN0 PTC patients is irrational. cN0 PTC patients with NSVCLNM are more likely to benefit from pCND. Defining the subset of cN0 PTC patients prone to NSVCLNM still remains a critical issue. Identification of the predictive factors of NSVCLNM in cN0 PTC is the routine method used to select this subset of patients. However, due to the low incidence of NSVCLNM, the positive predictive value of having 1 risk factor in the prediction of NSVCLNM in cN0 PTC was just 2.9%. Therefore, we attempted to assess the effect of coexistence of 2 or more risk factors for predicting NSVCLNM in cN0 PTC. We found that the sensitivity and specificity of having 3 or more risk factors for predicting NSVCLNM in cN0 PTC was 46.3% and 86.8%, respectively, which is similar with the efficacy of ultrasonography in detecting central LNM of PTC.^[53,54]^

The present study has some limitations. In our center, ipsilateral pCND was performed routinely for cN0 PTC, whereas bilateral pCND was performed if the lesions occurred in the isthmus or the malignant nodules were located in both lobes. Thus the extent of pCND included unilateral and bilateral clearance, which may have influenced the number and size of metastatic lymph nodes in the central compartment, since the prevalence of contralateral paratracheal LNM has been reported to be from 3.9% to 30.6% in unilateral cN0 PTC.^[3,48,51,55–59]^ Additionally, this was a retrospective study and the parameters such as tumor size and multifocal lesions were both determined through paraffin section pathological examination after surgery, which may have influenced the observed efficacy for predicting NSVCLNM before or during operation.

In conclusion, male gender, age ≤ 36 years, multifocal lesions, extrathyroidal extension, and tumor size >0.85 cm were considered as independent risk factors for NSVCLNM in cN0 PTC. PTC patients with 3 or more of the above parameters were likely to be associated with more benefits than potential risks from pCND.

## Author contributions

**Conceptualization:** Jian-Biao Wang.

**Data curation:** Jian-Biao Wang, Ya-Yu Sun, Liu-Hong Shi.

**Formal analysis:** Jian-Biao Wang, Ya-Yu Sun, Liu-Hong Shi.

**Methodology:** Jian-Biao Wang, Ya-Yu Sun.

**Supervision:** Lei Xie.

**Writing – original draft:** Jian-Biao Wang, Ya-Yu Sun, Lei Xie.

**Writing – review and editing:** Lei Xie.
